# “It is always HIV/AIDS and TB”: Home-based carers’ perspectives on epilepsy in Cape Town, South Africa

**DOI:** 10.3402/qhw.v11.30213

**Published:** 2016-06-01

**Authors:** Mpoe Johannah Keikelame, Leslie Swartz

**Affiliations:** 1 Primary Health Care Directorate, Faculty of Health Sciences, University of Cape Town, Cape Town, South Africa; 2 Department of Psychology, Stellenbosch University, Stellenbosch, South Africa

**Keywords:** Epilepsy, home-based carers, focus groups, religious beliefs, South Africa

## Abstract

The study highlights the complex cultural religious factors affecting epilepsy and a need for integrated home-based care services. Two focus group discussions exploring home-based carers’ (HBCs) perspectives on epilepsy were conducted using a semi-structured focus group interview guide, which was based on Kleinman's explanatory model framework. The audio-recorded data were transcribed verbatim, and a thematic analysis was done. The three main themes were epilepsy names and metaphors, religious beliefs about the cause and treatment of epilepsy, and HBCs’ perceived roles and strategies for engaging in epilepsy care. Findings provide some insights for research, policy, and practice.

There is a growing concern that in low- and middle-income countries (LMICs), the weak health system, poor access to basic needs, and lack of attention to social determinants affecting health of the marginalized population groups might affect the attainment of the Millennium Development Goals (MDGs) (Nxumalo, Goudge, & Thomas, 2013). Similarly, in South Africa, the health-care system is also reported to be ill-equipped to deal with health-care challenges (Levitt, Steyn, Dave, & Bradshaw, 2011)—and this has led to the reemergence of the deployment of community health workers (CHWs) as a strategy to improve access to health care through community outreach services (Nxumalo et al., 2013; Schneider et al. 2008). These CHWs have been recognized as a potential resource to overcome the shortage of human resources in a range of health-care settings (Van Ginneken, Lewin, & Berridge, 2010). Their services have been mainly through non-profit organizations (NPOs), which are funded by the government, and their role has been noticeable within the HIV/AIDS field (Van Pletzen, Zulliger, Moshabela, & Schneider, 2013).

Whereas the South African government is putting more emphasis on the reengineering of primary health care (PHC) and have a number of different types of CHWs estimated to be between 60,000 and 70,000, these workers have not yet received appropriate recognition (NACOSA, 2013). According to Swartz (2013), part of the difficulty with literature on these CHWs is that they are often regarded as a homogenous group without considering their differential experiences, roles, motivations, and understandings of health and health care. Another concern is that the CHW model has no standardized policy regulating their program and training (Haynes, Hunter, & Jassat, 2011).

Currently, the South African health system is faced with emerging non-communicable diseases (NCDs) in both rural and urban areas, which are characterized by poverty and marginalization (Mayosi & Benatar, 2014). Of great concern is that epilepsy is not reported as one of these emerging NCDs and may therefore not receive equal attention—yet it is among the six top NCDs which are managed in primary care settings (Lalkhen & Mash, 2015). In the provincial integrated NCDs audit report of the Western Cape Province, Cloete (2015) highlights that the prevalence of epilepsy is unknown—indicating a gap in research. The report further shows that more emphasis is on the biomedical aspects of epilepsy and not on the psychosocial aspects, which pose major challenges for people with epilepsy (PWE) and their carers (Bhalla et al., 2013).

There are very few studies looking at CHWs roles in epilepsy in South Africa. We are not aware of any CHW studies which explored home-based carers’ (HBCs) perspectives on epilepsy in an urban township in Cape Town. Our objective was to analyze and describe their subjective experiences and perspectives on epilepsy and to provide information that can be used to guide planning and development of appropriate interventions to improve epilepsy treatment and care.

## Methods

### Research design

We used an exploratory qualitative design using focus group method to answer the question: “How do HBCs, working in a low-income area of Cape Town, understand epilepsy, its management, and what is their potential role in this?” This method uses group interaction to produce data and insights that would be less accessible without the interaction found in the group (Ulin, Robinson, Tolley, & McNeill, 2002, p. 92). These lay carers are regarded as important sources from which everyday ideas about the illness, support, and advice are sought and first-hand experience of suffering are gained (Kleinman, 1980).

Our Focus group discussions (FGDs) interview guide was based on Kleinman's (1980) explanatory models of illness (EMs). These EMs constitute a way of understanding how people recognize an illness, explain it, and respond to it. In addition, these EMs are shaped and influenced by culture and are held by patients and their carers, and can provide personal and social meaning with regard to the illness experience. Using Kleinmans’ (1980) EMs would enable us to elicit HBCs perspectives and experiences on epilepsy on how they explain, recognize, and respond to the illness and its symptoms. These HBCs provided services for patients with other chronic illnesses, but not epilepsy (see Box 1). The study was approved by the four local health research ethics committees: the University of Cape Town, Stellenbosch University, the Provincial Department of Health, and City Health.

Box 1 **Example of HBCs FGD interview guide questions.**

From your point of view, what do people in your community call epilepsy? Are there other names that are used to refer to epilepsy? Why are they used and what do they mean?

What do people in your community think is the cause of epilepsy? Why do they think so?

What do you think is the cause of epilepsy? How does it affect the person having the illness?

How serious is epilepsy? What course do you think it takes?

What kind of treatment do you think people with epilepsy should receive? What outcomes do you expect from the treatment you mentioned?

What are the kind of difficulties that people with epilepsy have in your community? What are the reasons thereof?

What kind of things make it difficult for people to understand epilepsy?

What kind of actions can be taken to address the kind of things you mentioned?

Who should take action and why? What can enable such actions? What can make it difficult to take such actions?

What kind of role can you play in caring for people with epilepsy?

What are the main things that people fear most about epilepsy? What do you fear most about epilepsy?

Adapted from Kleinman (1980).

### Study setting

The setting is an urban township in Cape Town and is characterized by high rates of unemployment, illiteracy, and poverty, and has a population of about 52,401 (Lehohla, 2011). Although there is a national non-government organization (NGO) which supports PWE and their families, its services are currently unavailable in the study setting. There are other health services such as the local community health center (CHC), private and general practitioners, and a local clinic that does not render health-care services for NCDs (Naidoo & Irlam, 2005).

### Recruitment and sampling

We recruited 18 HBCs who were employed by the local NGO, which is registered with the South African Department of Labour and provides basic HBC services in the study setting. The organization is faith-based and its mission is to care for all regardless of faith background. A convenience and purposive sampling method was used for recruitment and inclusion. Prior to recruitment, MJK set up a meeting with HBCs and their manager to present the project proposal and purpose of the study to start the recruitment process and to gain informed consent. MJK was accompanied by a Xhosa-speaking female field assistant whose role was to assist in the interpretation of the informed consent and participant information regarding their responsibilities and rights to participate and to ensure their understanding of the information in order to make informed decisions and choices to participate. MJK can speak isiXhosa but it is not her first language—having the field assistant enabled accessibility to the participants on the basis of language. The informed consent and information leaflet that stated the purpose of the study, their right to participate or withdraw, and the importance of not divulging information were read by the field assistant. Time was given for questions and clarifications. They were all given signed copies of their informed consent forms after obtaining written consent and were requested to bring them along on the date of the FGD. Kroll, Barbour, and Harris (2007) report on the importance of asking focus group participants to read the consent documents and to sign them before discussions.

Because all 18 HBCs were willing to participate and that the acceptable group size was between 6 and 12 participants in FGDs (Kroll et al., 2007)—two separate groups were arranged for each group at their convenient times. These HBCs were included because they would be in a position to provide in-depth insights (Gerrish & Lacey, 2010) on the topic and also that they might have cared for PWE or patients with other chronic illnesses who might also have epilepsy.

### Data collection

Two FGDs were conducted by MJK on April 16, 2013, and April 30, 2013, at the HBCs’ workplace—which was their preferred venue—and consisted of nine participants each. Although they chose to be interviewed at their place of work, we cannot guarantee confidentiality and anonymity between the participants themselves. Reporting on measures to ensure confidentiality in focus groups, Powell and Single (1996) suggest measures such as asking focus group participants to sign a written declaration that they will not divulge any information, and giving them copies signed by the principal investigator and the participants with each of them retaining a signed copy of the document.

The focus group interview guide was constructed in English and translated and was used to collect the data. It was pre-tested and no changes were made. Permission to audio-record the discussion and to disseminate findings was sought and the roles of MJK and the note taker were clarified. The duration of each FGD was between 90 and 120 min. During the two FGDs, some individual participants asked specific questions such as, “How can a PWE who has other chronic illnesses (diabetes, asthma, cardiac) be cared for?” and were given time to discuss these questions in order to capture the responses, context, and content of the discussion and only proceeded to the next topic when the group felt that they had no additional input.

There were some participants who used “I” when they talked about their individual experience while others used “we” to include others. MJK would pose questions to determine if the issue was a collective or an individual perspective. There were instances where all participants expressed gestures such as “laughter” when they talked about the epilepsy metaphor “plucking the chicken.” MJK would check why they laughed at that point in order to clarify assumptions and misinterpretations. Because the FGD were held 2 weeks apart, MJK had time to read and to listen to the first audio-recorded discussion. This enabled MJK to follow up on some issues raised in the first FGD to gain a broader perspective.

MJK asked each participant to reflect on the process and their comments were audio-recorded, and these were completed as field notes by MJK after debriefing sessions with the note taker and were further expanded by the MJK for use in the data analysis process (Watt, 2007). Refreshments were served and each participant received a transport voucher of R20.00 (approximately two US dollars).

## Data analysis

The audio-recorded data from the two FGDs were transcribed verbatim from isiXhosa into English by a Xhosa-speaking language practitioner. Consistent with Braun and Clarke (2006) thematic analysis method, MJK read the two transcripts and listened to each audio-recorded discussion of the two data sets to gain a sense of what participants talked about. During this process of familiarity and immersion, MJK listened to each audio-recorded FGD data and simultaneously read each transcript word by word and line by line to ensure that the actual participants’ responses had been transcribed verbatim from isiXhosa into English, and to ensure that the transcribed data accurately reflected the perspectives of the group discussion and individual responses and used field notes to fill in any gaps in information (MacMillan, McKee, & Sadler, 2007). MJK used an inductive approach to identify common themes from the data as well as questions and topics that were raised by participants themselves (Twinn, 2000). MJK copied and pasted the coded sections of the two data sets into MS word two-column tables in order to modify, group, and regroup the themes to ensure that no code had been missed and that the described and named themes provide a clear sense of what each theme was about (Braun & Clarke, 2006).

Rigor was ensured by confirming findings with participants (Anney, 2014; Flick, 2006) and by meeting regularly with the second author to reflect on how our own assumptions, professional orientations and backgrounds might have influenced the data analysis and interpretation thereof (Adams, McCreanor, & Braun, 2013). We did not identify any new theme from the two data sets. We were thus satisfied that we had reached thematic saturation (Onwuegbuzie et al. 2009). We also used pseudonyms to protect the identity of participants.

## Results

A total of 18 Xhosa-speaking HBCs participated in the study. Out of these, 17 were females and 1 was a male. Their mean age was 41.1 years. Their years of practice ranged from 1 to 7 years with the mean years of practice being 3.4 years and they belonged to different religious organizations. Their demographic profile is provided in Table I.

**Table I:** HBCs FGD participants’ demographic profile.

HBC, *N*=18	Age	Religion	Education	No of years	Gender
C01	59	Methodist	Grade 12	7	F
C02	59	Universal	Grade 10	7	F
C03	53	Anglican	Grade 10	7	F
CO4	53	Methodist	Grade 12	3	F
C05	48	Methodist	Grade 12	1.5	M
C06	48	Zion	Grade 11	3	F
C07	46	Universal	Grade 12	3	F
C08	46	Universal	Grade 10	3	F
C09	42	Apostolic	Grade 10	3	F
C10	38	Methodist	Grade 10	3	F
C11	36	Anglican	Grade 12	3	F
C12	36	Apostolic	Grade 10	3	F
C13	35	Zion	Grade 10	3	F
C14	34	Methodist	Grade 12	2	F
C15	34	Anglican	Grade 12	3	F
C16	29	Ethiopian	Grade 12	3	F
C17	29	Roman	Grade 11	3	F
C18	25	Zion	Grade 10	1.5	F

Seven key themes emerged from the data: (i) names and metaphors referring to epilepsy; (ii) religious beliefs about the cause and treatment of epilepsy; (iii) views about marriage, driving, employment, and schooling; (iv) difficulties affecting access to treatment and care;(v) difficulties caused by the illness, (vi) fears about the illness and (vii) HBCs’ perceived role and strategies for epilepsy care. We report on three themes that are of central focus of this article: (i) names and metaphors referring to epilepsy; (ii) religious beliefs about the cause and treatment of epilepsy; and (iii) HBCs’ perceived role and strategies for engaging optimally in epilepsy care.

### Names and metaphors referring to epilepsy

Participants used different Xhosa names for epilepsy—*isifo sokuxhuzula* (illness of fitting), *ukuxhuzula* (fitting or fits), and *isifo sokuwa* (illness of falling). An interesting finding was the metaphor “plucking the chicken” (*ukuhlutha inkuku* in isiXhosa). We noted that most participants enjoyed discussing this metaphor, but we thought that their explanations highlighted the dramatic nature of the tonic–clonic seizure and how people perhaps behave or act toward a person during a seizure. We also thought that their explanations for the shaking of the chicken when it is being slaughtered were about presentation and recognition of symptoms during a tonic–clonic seizure. For example, three participants discussed the metaphor as follows:

Participant (P): I was going to say … others say to pluck the chicken …

(Laughter).

P: Yes, to pluck the chicken, because when you are plucking the chicken you pull those feathers so when you are about to kill it you see it shaking.

P: Also when you have already killed it, when you have cut the neck, it shakes

(Laughter).

P: So it's doing what is done by a person who fits. At that time it hasn't died completely. It's still going to die. It's still in the process of dying.

When participants were probed to explain why people use this metaphor, we found that they spoke about death and laughter which we thought were indicative of the seriousness of epilepsy (that people can die from epilepsy) and the stigma toward people living with the illness (laughter, joke, thing):

P: They think he will fit and die.

P: It's death and laughter.

P: I was saying another one makes a joke about that thing.

P: But really others fit and die.

### Religious beliefs about the cause and treatment for epilepsy

There was a lengthy discussion on religious beliefs about the cause of epilepsy and its treatment. We noted that HBCs had varied perceptions on the topic and how epilepsy is perceived by the community. For example, one participant said people hold the belief that epilepsy is caused by evil spirits:

P: They (people) believe that tradition thing that it is an evil spirit that causes you to fall (to have epilepsy).

One participant challenged this view and responded that the belief about evil spirits is not held by all people. However, the same participant stated that this belief is held by his or her family and that decisions about the choice of treatment are done by significant others.

P: It goes fifty-fifty (there are those who hold these beliefs and those that do not)… because at my home if I'm fitting they will say “Oh, an evil spirit”, and church, yes, because they believe that the church can heal me through prayers.

Another one explained that evil spirit is a “demon inside the person.” This participant affirmed the previous participants’ response that significant others play a role in the choice of treatment for a family member whose illness is believed to be caused by demons or evil spirits. The participant further stated that it is often very difficult to challenge these cultural beliefs probably because the participant might have noticed some PWE who had poor treatment outcomes on either western or religious treatments.

P: Yes sometimes that spirit is the demon inside you and then maybe your parents take you to this faith healing process… people start praying and praying and then all of a sudden now you start falling (having a seizure) because now that prayer has touched you (prayer triggered the seizures) and you sometimes vomit, then they say “Ah, it's gone out, it's gone out!” (The spirit is released). So you can't guarantee what is what (what treatment is best).

As the response below displays, another one supported the previous one's views about the challenges faced by HBCs in dealing with religious beliefs related to epilepsy. Although this participant spoke on behalf of other group members, he or she highlighted that the difficulty in addressing these cultural beliefs is because they are professed by influential and powerful religious leaders—and that people believe their treatment instructions that often result in poorly controlled seizures:

P: What concerns us is that they (Faith healers) say, “Don't take the tablets because you have been healed in the name of Jesus”. Then patients do not take the tablets (seizure medication) and then they start fitting again because they are not going to church.

In view of other participants’ responses, another one challenged the previous ones’ views probably because the participant might be having strong religious beliefs about the cause and treatment for epilepsy:

P: Like I believe that I'm going to be right because I go to church. I'm not going to drink my tablets because I believe in church. That's what I'm saying, I'm using my faith. By praying I'm going to get healed without tablets. I'm going to get healed. I'm not going to use the tablets. By faith I'm getting healed by the prayer – the power of prayer.

Another participant disagreed with the previous participant's views and reiterated that despite the former participant's personal religious beliefs about epilepsy treatment, it is important that the particular HBC should provide appropriate advice to patients under his or her care with regard to the importance of taking seizure medication. We were of the view that this participant was probably highlighting that HBCs should not impose their personal beliefs on those under their care—but that they must give appropriate advice:

P: No, you must tell them (those who believe that their epilepsy can be healed by prayer) to take their medications.

### HBCs’ perceived role and strategies for engaging optimally in epilepsy care

Participants were first asked how epilepsy is understood by their community. They all thought that there was a general lack of understanding of the illness because of too much focus on HIV/AIDS and TB.

It's always HIV, HIV… and yet there are lots of things that are worrying people and some people don't even know what causes epilepsy. It's only TB, HIV. People are concentrating on AIDS and TB.

When asked on how the lack of information and knowledge about epilepsy could be addressed, HBCs responded to the question as a collective and gave some examples of strategies to address the problem:

We could give talks at the taxi rank.

We can organize and do campaigns at churches, schools, train stations and give pamphlets.

We can do short plays – it's easier when they see it.

Another one highlighted the kind of health promotion activities that would be welcomed by community members:

Here in the community they do want to be educated through campaigns, door to door home visits giving pamphlets, education at school and road shows.

In addition to the above-named strategies and their awareness of the knowledge gap, they suggested that epilepsy could be integrated with other chronic illnesses and that PWE should be encouraged to educate others about their illness and to demystify the illness:

It's important to have health talks on different illnesses in health center … then there is also a health talk about epilepsy and then have one patient saying I'm having epilepsy but I am working, I'm not dependable on my family … educate everyone whether a family has a person with epilepsy or not so that they can be of help to the next person.

Regarding their role in epilepsy care, HBCs highlighted that they could provide basic counseling and referral through collaborative partnerships.

The other thing as we are working for our organization, we all have patients with epilepsy. We can counsel them. We can refer them to the Epilepsy NGO maybe they (Epilepsy NGO can come once a month to our organization. We can gather our patients to meet with them to get more information from them.

Another participant thought that it was important that HBCs should receive training on epilepsy in order to increase their understanding and to gain appropriate skills such as organizing educational campaigns, conducting support groups, and assisting with some tasks in the hospital.

I was going to say we must first get educated on epilepsy so that we can educate people in the community. Then we can organize support groups. We can also go to hospitals and see what we can do to help.

## Discussion

Our study aimed to describe and analyze HBCs’ perspectives on epilepsy in an urban township in Cape Town, South Africa—seventeen were women and the eighteenth one was a male. In South Africa, this type of work is done mainly by unpaid volunteers or by those who are fully employed with most being primarily women (Daniels, Clarke, & Ringsberg, 2012).

HBCs provided names that are commonly used to refer to epilepsy—*isifo sokuwa*, *isifo sokuxhuzula*, *ukuxhuzula*, and *fits* which were similar to those reported by Keikelame and Swartz (2013, 2015). However, we note from literature that these terms often refer to epilepsy as well as seizures. In Kilifi, in the coastal region of Kenya, different terms such as *Nyuni*, *Nyago*, *Nyama ya dzuka*, *Vitsala*, and *Kifafa* are used to refer to seizures—and these are associated with different causes (Mbuba et al., 2012). According to these authors, “when medical explanations fail to help patients to understand their condition, they are most likely to believe in culture-specific meanings of the condition and its cause” (Mbuba et al., 2012, p. 480). Therefore, lay explanations cannot be ignored even though they might not be scientific (Zhu, Liu, & Tardif, 2009).

Epilepsy stigma and stigmatizing names have been widely reported in literature (De Boer, 2010). Similar names such as “it” and “a thing” were also reported in South African studies on epilepsy by Keikelame and Swartz (2013, 2015). The most disturbing new finding was the metaphor referred to as “plucking the chicken.” According to Helman (1994), illness metaphors or clusters thereof become a way through which people express their fears or anxieties about the condition. From our interpretation, we thought the use of this metaphor is probably a way of expressing the perceived seriousness of the illness. We argue that although this may be interpreted as a form of stigma, it is an African expression which can enable understanding of the presentation of symptoms and how they are explained by witnesses in order to enable appropriate diagnosis. Poor witness description has been reported as a barrier to management of epilepsy (Keikelame, Hills, Naidu, De Sá, & Zweigenthal, 2012).

The prominent theme was about religious beliefs. We thought that this could be due to the fact that the HBCs’ organization is religious and that this might have accounted for its prominence. Noting from literature, Obeid, Abulaban, Al-Ghatani, Al-Malki, and Al-Ghamdi (2012) report that religious beliefs about epilepsy and spirit or demonic possession, or that an alien spirit has entered the individual, have been well documented. They have been held among different religious cultures such as the Greco-Roman, Judeo-Christian, Islamic, Hindu and Voodoo traditions (Cavanna, Cavanna, & Cavanna, 2010)—but there is very little research focusing on these aspects (Ismail, Wright, Rhodes, & Small, 2005).

An interesting finding was also on the faith healing process. HBCs explained that during this treatment, a PWE goes into a trance—and when they vomit, people believe that the spirit has left their body. This finding shows the powerful nature of faith healing. Reporting on this aspect, Truter (2007, p. 58) highlights that faith healing process is carried out by faith healers or prophets who believe that they possess the healing powers from God “through ecstatic states and trance-contact with a spirit which they refer to as ‘*umoya*’.” Therefore, healers and family members who hold these beliefs may discourage PWE to take seizure treatment. We were thus concerned about the extent to which these beliefs would influence care giving practices of HBCs who value them. The findings indicate a need for mapping these religious beliefs when designing interventions (Otte et al., 2013b) including interventions that promote cultural literacy (Zarcadoolas, Pleasant, & Greer, 2006).

HBCs identified the knowledge gap in epilepsy care which they thought was due to more focus on HIV/AIDS and TB. They were of the view that integrating HIV/AIDS and TB programs with other NCDs would be beneficial. This view has also been expressed by Levitt et al. (2011) and Oni et al. (2014) and highlighted examples of the WHO frameworks that can be used—but need to be evaluated for appropriateness to local context.

Furthermore, HBCs believed that they can engage optimally in epilepsy care through collaborative partnerships with the national organization for epilepsy and task-shifting. Studies on CBR engagement in epilepsy care such as those in Guinea-Bissau (Otte et al., 2013a), in rural India (Nizamie, Akthar, Banerjee, & Goyal, 2009), and in Kilifi, Kenya (Carter et al., 2012), revealed that these workers can contribute to improving community-based epilepsy care. Our participants suggested “task-shifting” which according to Ledikwe et al. (2013) involves delegating tasks to less specialized health workers. Successful outcomes thereof were reported in Cameroon (Kengne, Fezeu, Awah, Sobugwi, & Mbanaya, 2010). In South Africa, it was explored by De Wet, Wouters, and Engelbrecht (2011) in HIV/AIDS programs and they found that nurses engaged in tasks that could be delegated to CHWs. As a result, a new policy has been developed which authorizes CHWs to do tasks such as finger-prick HIV testing.

### Limitations

We acknowledge that our orientations, interest, positions, and backgrounds might have affected the interpretation and analysis of the data. MJK is an older African woman who has worked among marginalized Xhosa-speaking communities and her previous research has been on epilepsy. The second author (LS) is a White man who has worked for many years in the field of culture, health, and disability, but is very much a cultural outsider.

In terms of FGDs, Smithson (2000, p. 116) states that in FGD, individuals tend to provide “socially acceptable opinions” and that the discussion might also be dominated by individuals who have power. This might have been the case in our FGD because of their organizations’ mission and their varied experiences and years of training.

## Conclusion

Our study explored HBCs’ perspectives on epilepsy and their perceived role in epilepsy treatment and care in an urban township in Cape Town. Our findings show different names and metaphors used to explain epilepsy and a knowledge gap which they attributed to too much focus on HIV/AIDS and TB. They further provide a glimpse of the complex nature of religious factors impacting on epilepsy and that these religious factors may affect provision of health care when held by health care workers. Our data suggest a need for an integrated approach to home-based care services in general and interventions that promote cultural literacy to enable these workers to respond respectfully and appropriately to patients under their care. Although these findings cannot be generalized, they provide some insights for research, policy, and practice.
